# Topology and Size Optimization of Trusses by Bone Remodeling: Primary Force-Based Approach

**DOI:** 10.3390/biomimetics11030223

**Published:** 2026-03-21

**Authors:** Burak Kaymak

**Affiliations:** Department of Civil Engineering, Faculty of Engineering, Kütahya Dumlupınar University, 43100 Kütahya, Türkiye; burak.kaymak@dpu.edu.tr; Tel.: +90-274-443-4144

**Keywords:** truss, topology optimization, ground structure method, force method, primary force-based approach, bone remodeling, kinematic stability, Michell truss

## Abstract

This study presents an optimization tool inspired by bone remodeling principles to address the high computational costs of truss topology optimization. Additionally, a new structural analysis method based on primary forces is proposed to overcome the kinematic stability problem. The strategy developed to obtain the optimal topology optimizes the initial dense ground structure in two stages. In Phase I, unnecessary members in the system are filtered to determine the “primary candidate members”; in Phase II, the final topology is reached through this refined subset. The algorithm performs an effective search in the design space by simulating biological processes that link the rate of mass change in the bone matrix to mechanical stimuli. Numerical results demonstrate high accuracy, as shown by the analytical solution of the 2D Michell truss, with a difference of 1.02%. The results show high consistency with reference studies, providing, in some cases, alternative topologies with the same weight and stiffness as given in the benchmarks. The proposed method achieves significant improvements in computational efficiency, reducing processing times for larger systems by 10 to over 250 times compared to literature benchmarks.

## 1. Introduction

Topology optimization has been a focal point of research in engineering and mathematics disciplines since the early 20th century. This method enables designers to systematically determine the optimal material distribution and structural configuration within the design space, thereby meeting their target criteria. Examples of techniques developed and widely used for topology optimization include the evolutionary structural optimization (ESO) [1], bidirectional evolutionary structural optimization (BESO) [2], penalised isotropic material (SIMP) [3], and the ground structure method (GSM) [4].

In the structural optimization literature, Evolutionary Structural Optimization (ESO), developed as an alternative to gradient-based methods, envisages the gradual removal of elements with low stress levels from the structure based on the principle of “eliminating the weakest link” [1]. However, the ESO method does not allow removed elements to be reintroduced into the design domain, which limits the algorithm’s adaptation to topological changes. To overcome this limitation, the Bidirectional Evolutionary Structural Optimization (BESO) method allows both the removal of inefficient elements and the addition of material to regions with high sensitivity. Both ESO and BESO use a 0/1 integer approximation. However, this approach incurs high computational costs, especially for large-scale problems [5].

To overcome the difficulties encountered in the integer programming approach, the problem is transformed using the solid isotropic material with penalisation (SIMP) method, with continuous density design variables [3]. In the SIMP approach, the discrete (0/1, presence/absence) problem, which is quite difficult to solve, is converted into a continuous form that is easier to solve. The presence of the material indicates full density ($\rho_{j}=1$), while the absence of the material indicates voids ($\rho_{j}=0$). Defining density as a continuous variable in the range [0, 1] transforms the problem from discrete to continuous form. Gray regions, defined as intermediate values of density ($\rho_{j}=0.5$, etc.), cannot be produced; therefore, gray regions are penalised to obtain the optimal topology [6,7]. The SIMP approach is more of a material representation method than an optimization algorithm [3]. Therefore, it can be used in both mathematical and heuristic methods [8].

Although continuous medium topology optimization methods (SIMP, ESO/BESO) are effective in form-finding processes for complex geometries, the results obtained generally require a secondary interpretation process to convert them into discrete trusses during the manufacturing stage [7]. In contrast, GSM defines the design domain from the outset as a dense network of potential bar elements, thereby transforming the optimization problem directly into a bar placement problem. The ground structure method consists of systems in which bar elements are connected between all possible node pairs [4]. A situation where a bar element is connected from one node to another is defined as a fully connected ground structure [9]. One of the fundamental challenges in density-based approaches is the possibility that the mesh resolution may affect the resulting design. In these methods, numerical instabilities such as mesh dependency and checkerboard patterns may arise [10]. On the other hand, increasing the mesh resolution rapidly increases the problem size due to the increased number of equilibrium equations. This leads to increased memory and computation time [11]. Particularly in large-scale engineering structures, the ability to formulate stress and buckling constraints directly at the element level makes GSM a more realistic option than SIMP and similar density-based approaches in terms of structural efficiency and manufacturability [12]. For the reasons listed above, the ground structure method is used in this study to obtain the optimal topology.

The ground structure method offers a holistic approach that simultaneously optimizes both the topological configuration and the element cross-sectional areas in truss design. Both gradient-based and heuristic methods are reported in the literature for obtaining optimal truss designs. Numerous heuristic methods are used to obtain optimal truss designs. In recent years, some studies using heuristic methods to obtain the optimal designs of trusses include: Artificial Bee Colony Algorithm [13], Enhancing Particle Swarm Optimization Algorithm [14], Bacterial Foraging Optimization Algorithm [15], Improved Vibrating Particles System Algorithm [16], Quantum-based Harmony Search [17], Improved Stochastic Ranking Evolution Strategy [18], Improved Crested Porcupine Optimization Algorithm [19], Improved Chef-Based Optimization Algorithm [20] can be listed. These population-based algorithms, which are part of heuristic methods, generally perform well on small- to medium-scale problems where the number of design variables is limited [21]. In population-based methods, the initial population and parameter selection significantly impact the resulting optimal design [22]. On the other hand, the greatest weakness of these methods is the necessity to repeat the structural analysis for each individual in every iteration [8,23]. To achieve an optimal result, the population size must increase as the number of design variables increases [13]. In large-scale systems with a larger number of elements, this situation poses serious problems, such as high computational cost [21,24]. As the design space expands, the convergence rates of these methods decrease, leading them to get stuck in local optima, prompting researchers to seek more efficient approaches.

To reduce these computational costs and obtain more stable results in large-scale systems, physics-based approaches that directly utilise the structural response have gained importance, replacing iterative population searches. At this point, shape-changing energy density (SED)-based approaches used in topology optimization exhibit strong parallels with bone adaptation mechanisms in biological systems. According to the principle known as Wolff’s Law [25], bone tissue optimises itself under mechanical loads, increasing its density in high-stress (high SED) regions while undergoing degradation in unloaded regions. This entire process is called “remodeling” [26,27]. Zou et al. [27] mathematically modelled this biological process and demonstrated that changes in bone density are directly dependent on local SED values. Therefore, SED-based element deletion/insertion methods (ESO/BESO) used in engineering can be considered a numerical imitation of this biological optimization process that nature has been applying for millions of years [28]. Methods such as SED, SIMP, and ESO/BESO, which are widely used in the engineering literature, aim to obtain the optimal topology by varying the density within a given volume. The study by Jang et al. [28] compared the SED-based bone remodeling algorithm and structural topology optimization. This comparison demonstrated that the SED-based bone remodeling algorithm developed in biomechanics can be matched with the gradient-based topology optimization problem. Although these biomechanics-based optimization principles are generally addressed in the literature through changes in volumetric density, this study focuses on integrating this logic into systems composed of discrete elements. In this regard, the bone remodeling algorithm was adapted to trusses to use GSM to obtain the optimal topology. The bone remodeling algorithm in the literature attempts to obtain the optimal topology by changing the density or elastic modulus in the relevant volume according to the SED value in a specific volume [28,29,30,31]. In this study, however, as described in Section 2.2, instead of modifying the density or elastic modulus by considering the stress value for each bar, the truss member cross-sectional area ($A_{j}$) is updated. Although this differs from applications in the literature, it should not be considered a new method but rather a new approach that applies the basic principles to trusses.

Whether the method used is heuristic or gradient-based, the relationship between the external and internal forces of the structure for which the optimal topology is sought must be established in a manner that satisfies the compatibility conditions. Although the displacement method, which incorporates these relationships and conditions, is accepted as a standard analysis tool in topology optimization, singularity issues in stress-constrained problems [32] and numerical instabilities in low-density regions [6] are among its fundamental disadvantages. The root cause of the problem lies in the numerical instability of the structure rigidity matrix (*K*) resulting from the removal of elements necessary for stability [33]. To prevent this situation, many researchers do not allow section areas to be exactly zero [31,32,34]. However, in this case, situations arise where bars that are very close to zero but do not actually exist appear to ensure stability, and it is not possible to reach the true optimum value [32]. Both gradient-based methods and heuristic methods are affected by the same problem. To overcome the kinematic stability problem of trusses, Ozbasaran [35] has proposed a solution for planar trusses. However, this proposal is not valid for spatial trusses. Shahabsafa et al. [36] presented an approach that also guarantees kinematic stability in the size and topology optimization problem of trusses. The mixed-integer linear optimization (MILO) approach was used in the study. Although the trusses considered in the aforementioned study are not very large-scale (the largest structure contains 990 truss members), the study reports that some analyses were terminated due to the solver’s 1-day time limit.

These numerical instabilities encountered in approaches using displacement methods via the stiffness matrix demonstrate the importance of the formulation of fundamental equations in structural analysis. A solution to this chronic problem is proposed using an approach in which unknowns are defined in terms of forces. The main goal of this study is to combine the original Primary Force-Based (PFB) analysis approach with bone remodeling principles, creating a computationally efficient optimization framework that ensures kinematic stability in large-scale trusses.

The two-phase (Phase I and Phase II) optimization strategy proposed in this study contributes to the advancement of biomimetics by combining the principles of bone remodeling in biological systems with a novel force-based approach. This hybrid structure introduces a novel approach to density-based methods in the literature by applying the physical adaptation logic based on Wolff’s Law in the form of updating the cross-sectional areas of truss structure members. A key feature of the proposed analysis approach is that it preserves the primary structure to ensure kinematic stability, while only allowing the removal of redundant members. Furthermore, the transition between Phase I and Phase II refines the solution space using energy-dense node filtering. By offering high computational efficiency and stability in complex, highly indeterminate systems, this method demonstrates how biologically inspired adaptation can be effectively transformed into a robust engineering solution.

To achieve the stated goal, the following research tasks are identified and addressed:

- Developing the primary force-based analysis approach to provide guaranteed kinematic stability during analysis.
- Implementing a biomimetic optimization rule based on Wolff’s Law to update member cross-sections using stress-based stimuli adaptively.
- Establishing a two-phase strategy to address the non-removable nature of the primary structure members, ensuring a path to the final optimal topology.
- Validating the precision and computational efficiency of the proposed framework through benchmark problems compared with existing literature.

The remainder of this work comprises the following sections: Section 2.1 presents the ‘primary force-based’ analysis method developed specifically for this study. Section 2.2 details the adaptation of the bone remodeling algorithm, used as an optimization tool, for truss structures. Section 2.3 describes the filtering method for eliminating unnecessary elements and nodes in the initial ground structure at the end of Phase I. Subsequently, Section 3 shows the application results of the proposed two-phase solution strategy on numerical examples and the effect of the signal coefficient (*c*) parameter on the algorithm’s performance. Finally, the findings are evaluated in the discussion and conclusion sections.

## 2. Methodology

### 2.1. Primary Force-Based Structural Analysis of Trusses

The mechanical behavior of a structure is solved by establishing equilibrium equations at the nodes [37]. This equilibrium condition is expressed as in Equation (1):

(1) BF=P

Here, *F* represents the independent end forces (in this study, the forces on the truss structure members), *B* represents the coefficient matrix of the equilibrium equations, and *P* represents the external load vector at the node point. The coefficient matrix has as many rows as the degrees of freedom (*p*) and as many columns as the independent end forces (*m*).

In statically indeterminate structures, equilibrium equations alone are not sufficient for calculating the system’s unknowns; therefore, compatibility equations are required. These equations are presented together in Equation (2):

(2) $$GF=B^{T}x$$

Here, *G* denotes the stiffness matrix of the truss members, $B^{T}$ denotes the compatibility equation coefficient matrix, and *x* represents the displacement vector. In the equilibrium equations given in Equation (1), the bar forces (*F*) are defined as unknowns. In a stable structure, the equilibrium equations contain at least one group of independent columns equal to the number of degrees of freedom. These independent columns, which are the end forces, have general load-carrying properties and are called primary forces ($F_{p}$). The forces remaining in the vector of unknowns, other than the primary forces, are called redundant forces ($F_{r}$). Thus, the vector of unknowns (*F*) is divided into two groups: primary structure forces ($F_{p}$) and redundant forces ($F_{r}$). When this grouping is performed, both the equilibrium and compatibility equations are rearranged as shown in Equations (4) and (5). Various $B_{p}$ and $B_{r}$ matrices can be constructed depending on the selection of the redundant force sets. Different techniques exist for selecting the primary structure [38]. In this study, the primary structure was determined by applying Gauss–Jordan operations on the coefficient matrix of the equilibrium equations given in Equation (1).

(3) $$B_{p}F_{p}+B_{r}F_{r}=P$$

(4) $$G_{p1}F_{p}+G_{r1}F_{r}-B_{p}^{T}x=0$$

(5) $$G_{p2}F_{p}+G_{r2}F_{r}-B_{r}^{T}x=0$$

Here, the submatrices of the flexibility matrix *G* have been rearranged by changing their positions in both columns and rows according to the primary structure forces and the redundant forces, and are given below.

(6) $$G^{*}=\left[\begin{array}{cc} G_{p1} & G_{r1}\\ G_{p2} & G_{r2} \end{array}\right]$$

Equations (3)–(5) contain three types of unknown quantities: the primary structure unknowns ($F_{p}$), the redundant force unknowns ($F_{r}$), and the displacements (*x*). The matrix $B_{p}$, which is the coefficient matrix of the primary structure unknowns, is a matrix with as many rows as degrees of freedom and as many columns as degrees of freedom. Since the $B_{p}$ matrix is obtained as full rank after Gauss–Jordan operations, it is a p×p matrix whose inverse can be taken. During Gauss–Jordan operations, pivot searches were performed in the rows and column swaps were carried out. Column swaps were performed both in the equilibrium equations coefficient matrix (*B*) and in the flexibility matrix (*G*). The flexibility matrix $G^{*}$, with its rows and columns rearranged, is denoted by $G^{*}$. Since the rows of the flexibility matrix have been rearranged, row swaps must also be performed in the coefficient matrix ($B^{T}$) of the geometric compatibility equations. This yields the new form in Equations (3)–(5). The new form has p+m rows and p+m columns.

Equation (4) is multiplied by $-B_{r}^{T}{B_{p}^{T}}^{-1}$ and added to Equation (5), eliminating the displacements from Equation (9). To simplify the notation, the definition $B_{pr}^{T}=B_{r}^{T}{B_{p}^{T}}^{-1}$ has been introduced.

(7) $$B_{p}F_{p}+B_{r}F_{r}=P$$

(8) $$G_{p1}F_{p}+G_{r1}F_{r}-B_{p}^{T}x=0$$

(9) $$\left(-B_{pr}^{T}G_{p1}+G_{p2}\right)F_{p}+\left(-B_{pr}^{T}G_{r1}+G_{r2}\right)F_{r}=0$$

The flexibility matrix for truss structures is diagonal because it shows the relationship between the deformation ($\Delta_{j}$) and the bar force ($F_{j}$) ($\Delta_{j}=\frac{L_{j}}{A_{j}E_{j}}F_{j}$). Therefore, the submatrices $G_{p2}$ and $G_{r1}$ given in Equation (6) are zero matrices. $G_{p1}$ and $G_{r2}$ are also diagonal matrices.

(10) $$G_{p1}\left(i,j\right)=\left\{\begin{array}{cc} \frac{L_{j}}{A_{j}E_{j}},\; & i=j\\ 0,\; & i\neq j \end{array}\right.,\;\begin{array}{c} i=1,2,\ldots ,p\\ j=1,2,\ldots ,p \end{array}$$

(11) $$G_{r2}\left(i,j\right)=\left\{\begin{array}{cc} \frac{L_{\left(p+j\right)}}{A_{\left(p+j\right)}E_{\left(p+j\right)}},\; & i=j\\ 0,\; & i\neq j \end{array}\right.,\;\begin{array}{c} i=1,2,\ldots ,r\\ j=1,2,\ldots ,r \end{array}$$

Here, $E_{j}$ is the elastic modulus of bar *j*.

If adjustments are made for zero matrices, Equation (9) takes the following new form.

(12) $$\left(-B_{pr}^{T}G_{p1}\right)F_{p}+G_{r2}F_{r}=0$$

The matrix $G_{r2}$ in Equation (12) is a diagonal matrix composed of the stiffness coefficients of the redundant force members, and therefore its inverse can be taken. Equation (12) is multiplied by $-B_{r}G_{r2}^{-1}$ and added to Equation (7).

(13) $$\left(B_{p}+B_{r}G_{r2}^{-1}B_{pr}^{T}G_{p1}\right)F_{p}=P$$

The redundant force unknowns ($F_{r}$) in Equation (7) are also eliminated, leaving the primary structure unknowns ($F_{p}$) as the only group of unknowns. The coefficient matrix formed in Equation (13) is a p×p square matrix. However, it is not a symmetric matrix. If the system of linear equations given in Equation (13) is multiplied on the left by $B_{p}^{-1}$ and the transformation $F_{p}=G_{p1}^{-1}\Delta_{p}$ is performed, the new form of the system of equations is as follows.

(14) $$\left[G_{p1}^{-1}+B_{pr}G_{r2}^{-1}B_{pr}^{T}\right]\Delta_{p}=P^{*}$$

Here, the product $B_{p}^{-1}P$ is denoted by $P^{*}$. Since the matrices $G_{p1}^{-1}$ and $G_{r2}^{-1}$ are diagonal matrices for truss structures, they are symmetric matrices. On the other hand, since the $G_{r2}^{-1}$ matrix is multiplied by $B_{pr}$ on the left and $B_{pr}^{T}$ on the right, the coefficients matrix given in Equation (14) is symmetric.

The elements of the $G_{p1}^{-1}$ and $G_{r2}^{-1}$ matrices are defined in terms of the bar cross-sectional area, $A_{j}$, the elastic modulus $E_{j}$, and the bar length $L_{j}$ as follows. Therefore, there is no need to perform laborious operations to obtain the inverse matrix.

(15) $$G_{p1}^{-1}\left(i,j\right)=\left\{\begin{array}{cc} \frac{A_{j}E_{j}}{L_{j}},\; & i=j\\ 0,\; & i\neq j \end{array}\right.,\;\begin{array}{c} i=1,2,\ldots ,p\\ j=1,2,\ldots ,p \end{array}$$

(16) $$G_{r2}^{-1}\left(i,j\right)=\left\{\begin{array}{cc} \frac{A_{\left(p+j\right)}E_{\left(p+j\right)}}{L_{\left(p+j\right)}},\; & i=j\\ 0,\; & i\neq j \end{array}\right.,\;\begin{array}{c} i=1,2,\ldots ,r\\ j=1,2,\ldots ,r \end{array}$$

In Equation (12), the product $G_{p1}F_{p}$ defines the axial deformations of the primary members ($\Delta_{p}=G_{p1}F_{p}$), while $G_{r2}F_{r}$ represents those of the redundant members ($\Delta_{r}=G_{r2}F_{r}$). Consequently, redundant member deformations can be expressed in terms of the primary structure deformations, as shown in Equation (17).

(17) $$\Delta_{r}=B_{pr}^{T}\Delta_{p}$$

It was stated that the flexibility submatrix $G_{r1}$ for truss structures is a zero matrix. Equation (8), when multiplied by ${B_{p}^{T}}^{-1}$ and rearranged, yields the displacements (*x*) in terms of the deformations of the primary structure members as given in Equation (18).

(18) $$x={B_{p}^{T}}^{-1}\Delta_{p}$$

By solving the system of equations in Equation (14), the deformations of the primary members are calculated, followed by the deformations of the redundant members using Equation (17). The stresses of the members with known deformations are then obtained as follows.

(19) $$\sigma_{j}=\frac{E_{j}}{L_{j}}\Delta_{j},\;j=1,2,\ldots ,m$$

When the equilibrium, constitutive, and compatibility equations of the system are considered together, the unknowns consist of displacements (*x*) and member forces (*F*). When the force method is used, the unknowns are arranged as redundant forces ($F_{r}$) [38,39]. In contrast, in Equation (13) presented in this study, the unknowns are defined as the primary forces ($F_{p}$). Although these unknowns are converted into the deformations of the primary members in a later stage, the primary forces essentially constitute the main unknowns of the established set of equations. Because the analysis phase directly requires determining the primary forces, and the equations are built upon them, this proposed framework is called the “Primary Force-Based” (PFB) approach.

**Proof.**  Positive Definiteness of the PFB Coefficient Matrix

The relationship given in Equation (14) is a system of equations where the unknowns are the primary member deformations ($\Delta_{p}$). When this expression is multiplied by $\Delta_{p}^{T}$ from the left,

(20) $$\begin{array}{c} \underset{\Delta_{r}^{T}}{\underset{︸}{\Delta_{p}^{T}B_{pr}}}G{r2}^{-1}\underset{\Delta_{r}}{\underset{︸}{B_{pr}^{T}\Delta_{p}}}+\Delta_{p}^{T}G{p1}^{-1}\Delta_{p}=\Delta_{p}^{T}P^{*}\\ \Delta_{r}^{T}G_{r2}^{-1}\Delta_{r}+\Delta_{p}^{T}G_{p1}^{-1}\Delta_{p}=\Delta_{p}^{T}P^{*} \end{array}$$

is obtained.

In truss structures, the relationship between member force and deformation is defined as $F_{r}=G_{r2}^{-1}\Delta_{r}$ for redundant members and $F_{p}=G_{p1}^{-1}\Delta_{p}$ for primary members. Consequently, Equation (20) represents twice (2U) the total strain energy (*U*) stored in the structure:

(21) $$U=\frac{1}{2}\left(\Delta_{r}^{T}G_{r2}^{-1}\Delta_{r}+\Delta_{p}^{T}G_{p1}^{-1}\Delta_{p}\right)=\frac{1}{2}\left(\Delta_{r}^{T}F_{r}+\Delta_{p}^{T}F_{p}\right)$$

Since strain energy in a physical system is always a positive quantity (U>0), the coefficient matrix of the linear system of equations defined in Equation (14) is positive definite.    □

The symmetric and positive definite nature of the coefficient matrix ensures the preference for Cholesky decomposition, a stable and efficient method for solving the Equation (14) system.

The procedural steps described above using the primary force-based analysis approach are presented in two parts (Algorithms 1 and 2) to align with the strategy used in the remainder of the study. The preparation stage presented in Algorithm 1 consists of the steps of establishing equilibrium equations, determining the primary structure, and obtaining the modified load vector. These steps are solely related to the equilibrium equations and are independent of member cross-sectional areas. Therefore, executing this algorithm only once is sufficient. In Step 2 of Algorithm 1, the $B_{p}^{-1}$ and $B_{pr}$ matrices required in Algorithm 2 are obtained by applying Gauss–Jordan operations on the coefficient matrix (*B*).
**Algorithm 1** Preparation of the Primary Structure and Load Vector1:Establish the equilibrium equations▹ Equation (1)2:Determine the primary structure via Gauss–Jordan elimination
3:Calculate the modified load vector $P^{*}$▹$P^{*}=B_{p}^{-1}P$

The second part of the proposed structural analysis approach involves establishing the system of equations, calculating the unknowns using Cholesky decomposition, and calculating the member stresses and node displacements. Since the system of equations is formed based on the cross-sectional areas, these steps in Algorithm 2 must be executed the areas change. When the same structure needs to be analyzed repeatedly for different cross-sectional areas, executing only Algorithm 2 is sufficient.
**Algorithm 2** Primary Force-Based Analysis Algorithm1:Establish the system of equations using Equation (14)2:Solve for the primary unknowns ($\Delta_{p}$) using Cholesky decomposition3:Calculate the deformations of redundant members ($\Delta_{r}$) using Equation (17)4:Calculate member stresses ($\sigma_{j}$) using Equation (19).5:(Optional) Compute nodal displacements (*x*) using Equation (18)

When the cross-sectional area of any redundant member (*j*) becomes zero ($A_{p+j}=0$), the *j*-th diagonal element in the $G_{r2}^{-1}$ matrix also becomes zero ($G_{r2}^{-1}\left(j,j\right)=\frac{A_{p+j}E_{p+j}}{L_{p+j}}=0$). Consequently, in the product $B_{pr}G_{r2}^{-1}B_{pr}^{T}$ in Equation (14), no value related to member *j* is transferred to the coefficient matrix. This signifies the removal of that redundant member from the system. If the cross-sectional areas of all redundant members become zero (i.e., all redundant members are removed), the coefficient matrix in Equation (14) becomes a diagonal matrix containing only terms related to the primary structure ($G_{p1}^{-1}$). As long as the primary member areas are not allowed to be zero, the structure guarantees kinematic stability, regardless of whether redundant members are present.

### 2.2. Bone Remodeling Optimization Algorithm for Trusses

Living bone tissue undergoes continuous strengthening or weakening processes under the loads it is exposed to [25]. The entirety of these processes is referred to as “remodeling” [26,27]. The mathematical modeling of this biological process by Zou et al. [27] revealed that changes in bone density are directly dependent on the local SED value. Subsequently, Jang et al. [28] demonstrated a direct mathematical analogy between the SED-based bone remodeling algorithm and gradient-based topology optimization. Studies in the literature also indicate that bone remodeling simulation is based on the principle of updating the density or elastic modulus of a given volume according to the SED value in that volume [28,29,30,31]. In this study, however, the relevant biological process has been adapted to the topology optimization of truss structures consisting of discrete elements rather than a continuous volume. In line with the reasons listed in Section 1, the ground structure method was preferred to create the design space. In this methodology, the bone remodeling algorithm eliminates unnecessary bars in the design space, leading the structure to its ideal topology. Accordingly, the objective function of the minimum weight problem, which is the fundamental goal of the optimization process, is expressed as follows:

(22) $$minW=\sum_{j=1}^{m}\rho_{j}A_{j}L_{j}$$

Here, *W* represents the total weight, *m* represents the total number of bars in the truss structure, and $\rho_{j}$, $A_{j}$, and $L_{j}$ represent the unit volume weight, cross-sectional area, and bar length of bar *j*, respectively. The design constraints that the objective function in Equation (22) must satisfy for the optimization problem are given below:

(23) $$\begin{array}{c} \sigma_{j}^{l}\leq \sigma_{j}\leq \sigma_{j}^{u}\\ A^{l}\leq A_{j}\leq A^{u} \end{array}$$

Here, *j* denotes the bar number, and $\sigma_{j}$ denotes the stress value occurring in bar *j*. $\sigma_{j}^{l}$ and $\sigma_{j}^{u}$ are the lower and upper limit stress values for bar *j*, respectively. $A^{l}$ and $A^{u}$ represent the lower and upper limits of the cross-sectional area of bar *j*, respectively. It is assumed that the lower stress limit is always less than zero ($\sigma_{j}^{l}<0$) and the upper stress limit is always greater than zero ($\sigma_{j}^{u}>0$).

In continuum models, this optimization process is typically performed using SED threshold values. In this study, the stress ratio method [40] was used, which is appropriate for the nature of truss structures. In a truss member subjected to axial force, the strain energy density is directly proportional to the square of the axial stress ($\psi =\sigma^{2}/2E$). Therefore, driving the optimization through stress ratios is mathematically equivalent to a local metabolic response to energy stimuli, providing a discrete biomimetic adaptation of Wolff’s Law. Furthermore, Klarbring and Torstenfelt [41] demonstrated that bone remodeling formulations and specific gradient-based topology optimization approaches are mathematically equivalent, thereby connecting biological adaptation to numerical optimization within a unified framework.

The methodology adopted in this study updates cross-sections by deriving a rescaling factor from the ratio of current stress to allowable stress, reflecting how bone optimizes its mass in response to mechanical loading. This approach effectively applies the biological ability to optimize mass to discrete structural elements. In this process, the signal functions representing the mechanical stimulus are expressed by Equations (24) and (25):

(24) $$s_{j}^{u}=\frac{\sigma_{j}}{\sigma_{j}^{u}}-1\leq 0$$

(25) $$s_{j}^{l}=\frac{\sigma_{j}}{\sigma_{j}^{l}}-1\leq 0$$

Here, $s_{j}$ represents the signal magnitude produced by bar *j*. A signal value greater than zero ($s_{j}>0$) means that the stress limits in the relevant element have been exceeded and that the cross-sectional area must be increased to ensure structural safety. This situation triggers osteoblast activity, promoting new bone formation. Conversely, a signal value below zero ($s_{j}<0$) indicates that the element is operating below capacity and that the cross-sectional area can be reduced to ensure efficiency; this process is equivalent to osteoclast activity, the destruction mechanism in bone tissue.

The structure reaches biological homeostasis (equilibrium) when the signal values of all elements approach zero. As given in Equation (26), the maximum signal value is determined as the design parameter of the relevant bar.

(26) $$s_{j}=max\left(s_{j}^{l},s_{j}^{u}\right)$$

According to Equation (26), the maximum signal value ($s_{j}$) determined for bar *j* is used as the fundamental parameter in calculating the new cross-sectional area of that element. The new cross-sectional area value is obtained via Equation (27), where the current area is revised according to this signal:

(27) $$A_{j}^{t+1}=A_{j}^{t}\left(1+c\cdot s_{j}\right)$$

Here, $A_{j}^{t}$ and $A_{j}^{t+1}$ represent the cross-sectional areas of the *j*th bar in the current and subsequent iterations, respectively. The variable *c* is a coefficient that regulates the signal level based on the principle of bone remodeling. In this biological process, mechanical stimuli drive gradual changes in bone mass [26]. These changes occur through slow-acting cellular pathways that adapt the structure over time [26].

In parallel with this biological reality, the *c* coefficient is introduced to simulate the speed of signal-level adaptation to the structure and to control numerical convergence. If the coefficient *c* is zero, cross-sectional area updates are inhibited. A value less than zero would result in the inversion of the generated signal, causing the structure to respond in direct opposition to the biological requirements, which is physically undesirable. In addition, multiplying the generated signal by a value greater than 1 would lead to excessive updates, contradicting the gradual nature of biological adaptation. For these reasons, the coefficient *c* must be in the range 0<c≤1. Although maintaining a high signal level increases the convergence speed, it can cause the solution to get stuck in local optima, especially in complex truss structures. Similar to the inherent slowness of the bone remodeling process, keeping the *c* value somewhat low reduces the convergence speed but ensures that the algorithm tends towards lighter designs.

The signal magnitude $s_{j}$ can take values in the range [−1,+∞] according to Equations (24) and (25). However, to ensure that the new cross-sectional areas to be used in the next step remain within the lower and upper limits of the cross-sectional area ($A^{l}\leq A_{j}^{t}\left(1+cs_{j}\right)\leq A^{u}$), the signal magnitude is constrained by the lower and upper limits given by Equations (28) and (29) ($s_{j}^{l}\leq s_{j}\leq s_{j}^{u}$):

(28) $$s_{j}^{l}=\frac{A^{l}-A_{j}^{t}}{cA_{j}^{t}}$$

(29) $$s_{j}^{u}=\frac{A^{u}-A_{j}^{t}}{cA_{j}^{t}}$$

The optimization process begins with an initial design created by assigning the upper limit values ($A_{j}^{u}$) to all cross-sectional areas. The primary structure is then determined using Gauss–Jordan elimination. At this stage, the bars are reordered such that the first *p* represent the primary structure and the remaining *r* represent the redundant forces (m=p+r). Subsequently, the lower limit cross-sectional areas of the redundant force members are set to zero ($j>p\Rightarrow A_{j}^{l}=0$). In comparison, the lower limit cross-sectional area of the primary structure members is set to the initial lower limit ($j\leq p\Rightarrow A_{j}^{l}=A^{l}$). This prevents the removal of the primary forces from the system, ensuring the stability of the structure. As a result of the structural analysis performed in each iteration, the bar stresses are calculated, and the feasibility of the current design is checked. If the stress constraints are violated, the signal value for the relevant bar becomes positive. The total constraint violation for iteration *t* is expressed as the sum of the positive signal values, as shown in Equation (30):

(30) $$violation=\sum_{j=1}^{m}max\left\{0,s_{j}\right\}$$

Next, the cross-sectional areas to be used in the next iteration are updated based on the calculated signal values for each bar. If any redundant force member cross-sectional area falls below the area lower limit value ($A_{j}<A^{l}$), the redundant force is removed from the system. Structural analysis is repeated for the updated design to perform a feasibility check. If the obtained design satisfies the constraints (i.e., is feasible) and the relative error in the structural weights across successive iterations is less than the predefined error tolerance ($\varepsilon_{w}$), the algorithm terminates. If these convergence criteria are not met, the process is repeated by returning to Step 6. The pseudocode for the proposed bone remodeling algorithm, defined by the objective function and constraints, is presented in Algorithm 3.
**Algorithm 3** Bone Remodeling Algorithm for Trusses1:**if** j≤p **then** $A_{j}^{l}\leftarrow A^{l}$ **else** $A_{j}^{l}\leftarrow 0$   ▹j≤p: Primary; else: Redundant forces2:$W^{new}\leftarrow$ Calculate the structural weight using Equation (22)3:Perform structural analysis using Algorithm 24:t←05:**repeat**6:    t←t+17:    $W^{old}\leftarrow W^{new}$8:    $s_{j}^{l},\;s_{j}^{u}\leftarrow$ Calculate signal limits using Equations (28) and (29)9:    $s_{j}\leftarrow$ Calculate the signal values using Equation (26)10:    $A_{j}\leftarrow A_{j}\left(1+c.s_{j}\right)$ Update cross-sectional areas11:    **if** j>p **and** $A_{j}<A^{l}$ **then** Remove bar *j*12:    $W^{new}\leftarrow$ Calculate the structural weight using Equation (22)13:    Perform structural analysis using Algorithm 214:    violation← Calculate the violation value using Equation (30)15:**until** $t\geq t_{max}$ **or** ($violation\leq \varepsilon_{v}$ **and** $\frac{|W^{new}-W^{old}|}{W^{old}}\leq \varepsilon_{w}$)16:The minimum weighted feasible design obtained is reported

### 2.3. Topological Filtering and Refined Ground Structure Generation

The analysis method proposed in Section 2.1 is based on determining the primary structure. However, since the optimal topology is initially unknown, the process of determining the primary structure by performing a full pivot search among the bars connecting the nodes forming the ground structure at the first level has been preferred. Using the obtained primary structure, the bone remodeling algorithm provided in Section 2.2 is employed to identify the necessary bars and determine their cross-sectional areas. These operations are defined as Phase I. The minimum cross-sectional area of bars in the primary structure must not be set to zero. Therefore, no primary structure member is removed from the system. This strategy also prevents nodes from being removed. This specific constraint is intentionally applied throughout the optimization phase to ensure kinematic stability.

At the end of the phase, there are nodes to which bars connected are zero bars or bars carrying very low forces. These bars and nodes should not be in the optimal structure. The primary structure selected at the beginning of Phase I necessitates this situation. However, when Phase I ends, many bars that should not be in the optimal structure have been removed from the system.

To achieve the optimal topology, it is necessary to identify the nodes and bars that are not required, i.e., to reconstruct the ground structure using the findings of Phase I. This section describes the calculation strategies used to achieve this objective.

#### 2.3.1. Identification of Energy-Dense Nodes

The nodal selection strategy assumes that strain energy is concentrated at junctions [42]. The nodal energy density $u_{i}$, representing the percentage of strain energy at node *i*, is defined as:

(31) $$u_{i}=\frac{\frac{1}{4}\sum_{j=1}^{q}F_{j}\Delta_{j}}{U}$$

where *U* is the total strain energy of the structure, *q* is the number of connected bars at node *i*, and $F_{j},\Delta_{j}$ denote the internal force and axial deformation of the *j*-th bar, respectively. The nodes are sorted in descending order according to their energy contribution. A priority list is then formed by selecting nodes until the cumulative energy contribution ratio ($\gamma_{cum}$) reaches 0.97. This threshold ($\gamma_{cum}=0.97$) preserves 97% of the total strain energy while filtering out nodes with minimum contribution. Although this value may be adjusted based on the problem’s characteristics, including the entire energy distribution, it can result in unnecessarily complex topologies.

#### 2.3.2. Nodal Refinement and Member Filtering

The designation of “primary candidate members” (PCM) and the subsequent refinement of the energy-dense node set are performed via Algorithm 4. This algorithm filters connected bars based on local energy averages ($\eta_{ratio}=0.03$). The $\eta_{ratio}$ acts as a local filter to remove bars with minimum contribution to local energy averages. While this limit can be tuned for different cases, including all members, it can lead to redundant connectivity in the final topology, which may adversely affect the decision on the actual necessity of the associated nodes.
**Algorithm 4** Primary Candidate Member Selection and Nodal Refinement1:**Input:** Nodal energies $u_{i}$ (Equation (31)), Threshold ratio $\eta_{ratio}=0.03$2:**Output:** Primary Candidate Member Set $M_{primary}$ and Refined Energy-Dense Nodes $N_{ED}^{*}$   **Global Nodal Filtering**3:Sort nodes based on $u_{i}$ and select initial subset $N_{ED}$ where $\sum u_{i}\geq 0.97$4:Initialize $N_{ED}^{*}\leftarrow N_{ED}$   **Local Adaptive Filtering and Nodal Validation**5:**for** each $Node\in N_{ED}$ **do**6:     ${\bar{u}}_{t}\leftarrow \frac{1}{q}\sum_{j=1}^{q}u_{bar,j}$▹ Average energy of connected bars7:     Filter incident bars where $u_{bar,j}\geq \eta_{ratio}\times {\bar{u}}_{t}$ into set *V*8:     **if** Number of unique directions in V≥2 (evaluated via Equation (32)) **then**9:   Add *V* to $M_{primary}$10:     **else**11:   $N_{ED}^{*}\leftarrow N_{ED}^{*}\setminus \left\{Node\right\}$▹ Discard node12:   Merge collinear bars in *V* into single continuous members13:     **end if**14:**end for**   **Bar Fragmentation**15:Split overlapping bars at incident $N_{ED}^{*}$ locations to ensure connectivity16:**return** $M_{primary},N_{ED}^{*}$

A critical feature of this process is the merging of collinear bars when a node is discarded. If a node fails to provide at least two unique directions—evaluated via the collinearity criterion in Equation (32)—it is removed from the set $N_{ED}^{*}$. To maintain structural continuity, the incident bars at that location are merged into a single continuous member, preventing the formation of unnecessary nodes.

#### 2.3.3. Geometric Robustness and Bar Fragmentation

In the reconstructed ground structure, a geometric check identifies and fragments bars passing through energy-dense nodes. The criteria for collinearity and projection are defined as:

(32) $$\left\{\begin{array}{cc} {\left|\vec{AP}\times \vec{AB}\right|}^{2}\leq \delta^{2}{\left|\vec{AB}\right|}^{2} & \left(\mathrm{Collinearity}\right)\\ \epsilon <\frac{\vec{AP}\cdot \vec{AB}}{{\left|\vec{AB}\right|}^{2}}<1-\epsilon & \left(\mathrm{Projection}\right) \end{array}\right.$$

**Figure 1:**
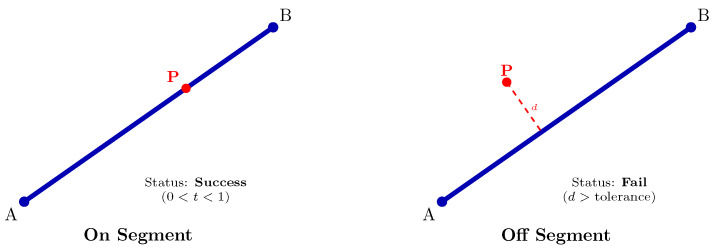
Geometric check.

The physical interpretation of these criteria, which ensures that fragmentation occurs only when a node is strictly within the interior of a bar segment, is illustrated in Figure 1.

#### 2.3.4. Refined Ground Structure Generation for Phase II

Based on the findings from Phase I, energy-dense nodes are identified using Algorithm 4. The same algorithm also determines the “primary candidate members”. Energy-dense nodes define the nodes of the structure to be created in Phase II. However, it cannot be guaranteed that the structure formed solely by the “primary candidate members” will ensure kinematic stability. Therefore, a fully connected ground structure is created among the energy-dense nodes. In the obtained structure, bars passing through the node (without connecting) are identified using Equation (32) and removed from the system. With this strategy, there are no overlapping bars in the created structure.

When determining the primary structure for Phase II, the bars identified at the end of Phase I as “primary candidate members” are given priority. In the pivot search operation, a full search is performed in the columns of the “primary candidate members” within the equilibrium equations established for energy-dense nodes. If no pivot is found, a pivot is selected from other columns. It is expected that the majority of the primary structure created with this strategy will be chosen from the “primary candidate members”. Bars included in the primary structure that are not “primary candidate members” will be included to ensure kinematic stability.

During the execution of the bone remodeling algorithm for Phase II, cross-sectional areas can be set to a starting point consistent with the findings obtained at the end of Phase I. This preference also helps the optimization algorithm reach the result with fewer iterations. However, to avoid a biased preference, all bar cross-sectional areas in Phase II are started with the maximum cross-sectional area ($A^{u}$), just as in Phase I.

#### 2.3.5. Illustrative Example of the Filtering and Refined Ground Structure Generation Process

This subsection provides a step-by-step illustrative example of obtaining the refined ground structure to be established after Phase I.

**Figure 2:**
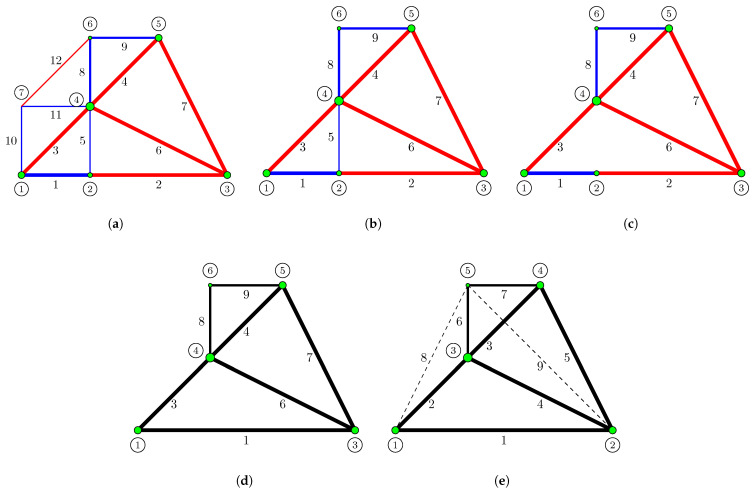
Illustrative example of the filtering and refined ground structure generation process (red lines: redundant members; blue lines: primary members; black lines: refined structure): (**a**) Initial energy-dense node set. (**b**) Removal of non-energy-dense node 7 and associated members. (**c**) Removal of low-energy member 5. (**d**) Removal of topologically unnecessary collinear node 2 and identification of the PCM list. (**e**) Final generation of the refined ground structure with updated numbering.

Upon completion of Phase I, energy-dense nodes (green nodes) are identified according to Steps 3 and 4 of Algorithm 4 (Figure 2a). It is observed that node 7 is not designated as an energy-dense node. In this case, node 7 and its connected bars (10, 11, and 12) are removed from the system, resulting in the structure shown in Figure 2b. However, there are still nodes and bars that must be removed for structural integrity and optimization efficiency.

Steps 5–14 in Algorithm 4 perform this refinement process. The bar numbered 5, seen in Figure 2b, has a low strain energy ($u_{bar,5}$) despite being located between the energy-dense nodes numbered 2 and 4. Since the energy of this bar does not meet the criterion calculated based on the average energy of the nodes to which it is connected (${\bar{u}}_{t}$) and the defined threshold value ($\eta_{ratio}=0.03$) ($u_{bar,5}<\eta_{ratio}\times {\bar{u}}_{t}$), bar 5 is removed from the system (Figure 2c).

After bar 5 is eliminated, a scan is performed on the energy-dense nodes (Step 8) to check local kinematic stability. As seen in Figure 2c, node 2 is now only the intersection point of two bars lying in the same direction (collinear). This indicates that the node is topologically unnecessary and cannot represent a degree of freedom on its own. Therefore, node 2 is removed, and bars 1 and 2 are merged into a single element (Figure 2d). When a similar check is performed for nodes 1, 3, 4, 5, and 6, local kinematic stability is preserved because it is found that at least two bars that are not collinear are connected to these nodes. This form obtained in Figure 2d constitutes the list of “primary candidate members” for Phase II. In 3D systems, local kinematic stability requires that at least three bars be connected to a node in different directions.

As mentioned in Section 2.3.4, it is not guaranteed that the structure formed solely by “primary candidate members” will provide kinematic stability. Therefore, as described in the relevant subsection, bars that are not among the “primary candidate members” but are required between energy-dense nodes are included in the system (Figure 2e). Node and bar numbers are reorganized. The newly generated ground structure does not contain overlapping elements; thus, an element connecting nodes 1 and 4 is not defined as it would pass through node 3, thereby avoiding redundant overlapping segments. Elements 1 to 7 are labeled as “primary candidate members” and serve as priority elements during the primary structure determination process in Phase II.

### 2.4. Overall Optimization Scheme

**Figure 3:**
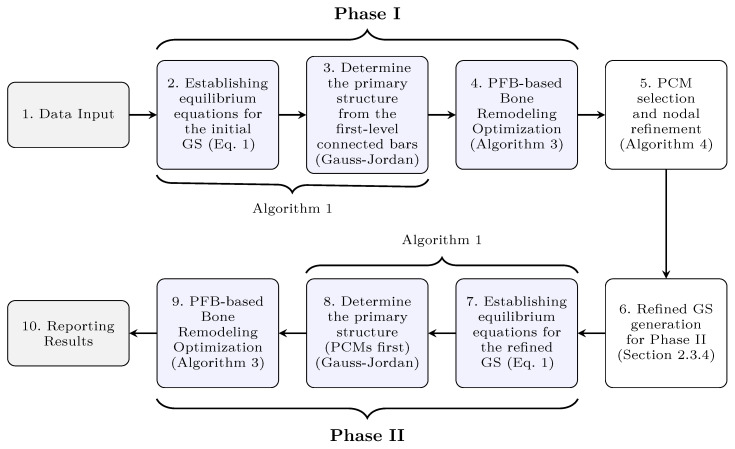
Flowchart of the two-phase optimization framework.

The individual components of the proposed methodology—namely, the PFB analysis, the bone remodeling optimization, and the filtering strategy—are integrated into a computational process. Figure 3 illustrates the overall optimization scheme, showing the interaction between these stages.

After establishing the equilibrium equations of the initial ground structure (Step 2), the primary structure is determined using Gauss–Jordan elimination among the first-level connected bars (Step 3). For the determined primary structure, the cross-sectional areas of the primary members are assigned as the minimum cross-sectional area ($A^{l}$). By contrast, the lower limit for redundant members is set to zero, ensuring that only redundant members can be removed from the system and maintaining kinematic stability throughout the process. The topology and size are then optimized using the PFB analysis tool coupled with the bone remodelling algorithm (Step 4). These steps constitute Phase I.

Using the results obtained at the end of Phase I, the energy-dense nodes and the list of “primary candidate members” (PCM) are identified via Algorithm 4 (Step 5). A refined ground structure is then generated based on the energy-dense node list (Step 6), representing a subset of the initial ground structure. The equilibrium equations for the refined ground structure are established (Step 7), and the refined primary structure is determined by giving priority to the members in the PCM list (Step 8). Consistent with Phase I, primary members are assigned $A^{l}$, and the lower limit for redundant members is set to 0. The final topology and size optimization is conducted through the integrated PFB and bone remodeling approach (Step 9), which completes Phase II. Finally, the optimized results are reported (Step 10).

## 3. Numerical Examples

To demonstrate the effectiveness of both the proposed PFB approach and the bone remodeling algorithm on the truss structure size and topology optimization problem, four example problems are examined. The results obtained are compared with those in the literature. In the bone remodeling algorithm, it was assumed that all bars existed in the system at the initial stage, as in the ESO [1] algorithm. Additionally, all bars are assigned the maximum cross-sectional area value ($A_{j}=A^{u}$) at the initial stage. Since the bone remodeling algorithm is not population-based, consistently utilizes a fully valued initial point, and does not incorporate any random operators within its mathematical structure, the results are independent of randomness.

To determine the effects of the signal coefficient *c* proposed in this study for the bone remodeling algorithm on convergence, 11 different values (c∈{0.01, 0.1, 0.2, 0.3, 0.4, 0.5, 0.6, 0.7, 0.8, 0.9, 1.0}) are tested. The results are shared in the relevant example-problem subsections.

In the structural visualizations, primary force members are represented in blue, while redundant members are colored red.

The structural analyses presented in this study were performed using specialized software developed by the author in C++. The software was designed without including any multithreading library. All calculations were run on a computer equipped with an Intel(R) Core(TM) i7-12700H (4.70 GHz) processor (Intel, Santa Clara, CA, USA) and the Ubuntu Linux operating system.

The control parameters used in the optimization process are summarized in Table 1. The algorithm is designed to terminate when the relative change in structural weight satisfies at a feasible point, or when the maximum number of iterations ($t_{max}$) is reached. It should be noted that $t_{max}=500,000$ is a safety limit to ensure termination in the event of non-convergence; in all numerical examples investigated, the algorithm converged before reaching this threshold. Detailed explanations of the cumulative energy contribution ratio ($\gamma_{cum}$) and the local energy averages ratio ($\eta_{ratio}$) are provided in Section 2.3. Furthermore, the bone remodeling algorithm performs only a single structural analysis per iteration. Feasibility is ensured when the sum of normalized positive constraint violations, as defined in Equation (30), remains within the threshold $\varepsilon_{v}$.

### 3.1. 12-Node Truss Problem

**Figure 4:**
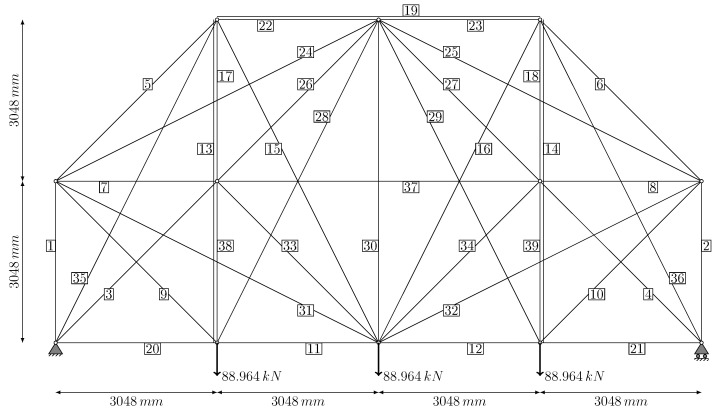
The 12-node ground structure bar connections, node, support and loading condition (member 13 overlaps 17 and 38; member 14 overlaps 18 and 39; member 19 overlaps 22 and 23).

The first example problem is a planar truss structure with 12 nodes and 39 members, selected as a test case to obtain results comparable to similar structural optimization studies in the literature. The members forming the structure and their associated nodes are shown in Figure 4. While this problem is frequently solved in the literature by exploiting its symmetry to simplify the search, the optimization in this study is conducted without symmetry exploitation. The technical parameters of the problem are presented below [33,43,44]:

Elastic modulus: E=68,948 MPa;

Weight density: ρ=27.14 kN/m^3^;

Allowable stress: $\sigma_{a}=137.90$ MPa;

Area: A=32.258to1451.61 mm^2^.

**Figure 5:**
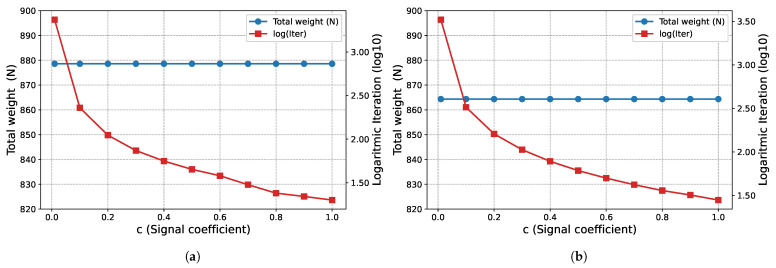
Influence of parameter c on the optimal weights and the required number of iterations in the 12-node truss: (**a**) Phase I; (**b**) Phase II.

The effect of the signal coefficient *c* on algorithm performance is presented in Figure 5. In this example problem, the total weights obtained at the end of Phase I (W=878.582 N) and Phase II (W=859.213 N) remain constant for all *c* values examined. On the other hand, it is observed that the number of iterations required for convergence decreases logarithmically as the *c* value increases.

**Figure 6:**
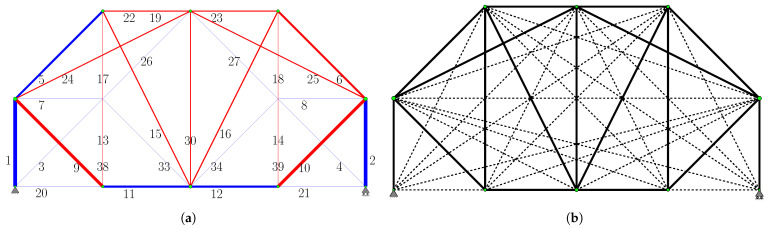
Results for the 12-node truss: (**a**) topology at the end of Phase I (blue: primary members; red: redundant members; member 13 overlaps 17 and 38; member 14 overlaps 18 and 39; member 19 overlaps 22 and 23); (**b**) generated ground structure for Phase II (shown in black).

The topology obtained at the end of Phase I is shown in Figure 6a. The system, which initially had 39 bars, consists of 32 bars: the primary force members (degree of freedom p=21) and 11 redundant force members. Seven of the redundant force members have been removed from the system. The green nodes in the figure represent the “energy-dense” nodes.

The ground structure reconstructed for Phase II is presented in Figure 6b. This configuration comprises 17 “primary candidate members” identified at the conclusion of Phase I (solid lines), supplemented by 21 additional members (dashed lines) to guarantee kinematic stability in accordance with the general strategy proposed in this study. Notably, members 20 and 21, which are essential for kinematic stability, were not among the “primary candidate members”; however, they were automatically incorporated into the primary structure by the algorithm during the Gauss–Jordan process for Phase II. Consequently, the Phase II optimization process begins with an initial ground structure of 38 bars, ultimately yielding the final 19-bar topology.

**Figure 7:**
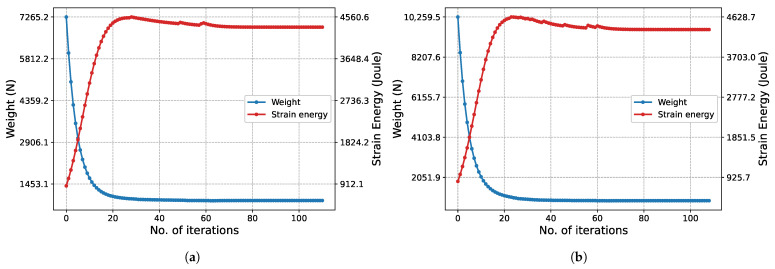
Convergence history of structural weight and strain energy for the 12-node truss: (**a**) Phase I; (**b**) Phase II.

The structural weight and strain energy change obtained in each iteration during the execution of the algorithm are shown in Figure 7. Initially, since all bars have the maximum cross-sectional area, the structural weight is at its maximum. In this state, the structure is at its most rigid state, so the strain energy is at its lowest possible level. As iterations increase, the cross-sectional areas decrease, reducing the structural weight while the rigidity decreases. This situation raises the strain energy. In Phase I (Figure 7a), it is observed that the strain energy reaches its peak at approximately the 20th iteration and then decreases. For the same iterations, the slope of the structure weight graph is seen to flatten. This situation is due to increased stress, despite the slight decrease in volume. In the subsequent process, both the structural weight and the strain energy change minimally until the final iterations. Similar behavior is also observed in the graph for Phase II (Figure 7b).

**Figure 8:**
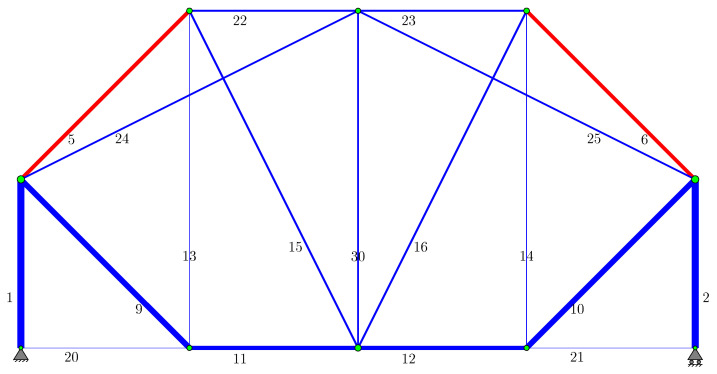
Final topology for the 12-node truss after Phase II optimization (blue: primary members; red: redundant members).

**Figure 9:**
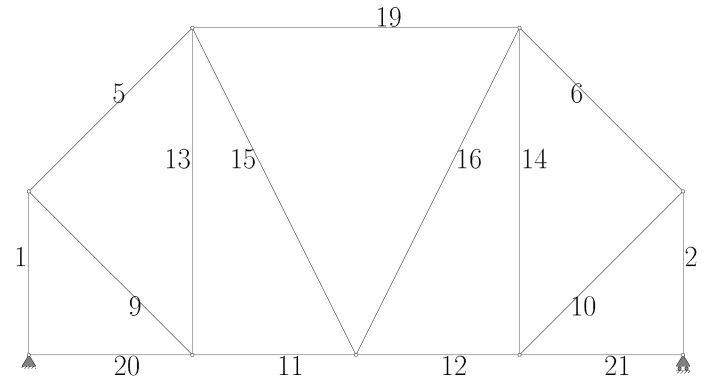
The reference truss topology proposed by Shakya et al. [33], Wu and Tseng [44].

The final topology obtained at the end of Phase II for the signal coefficient c=0.20 is shown in Figure 8, and the cross-sectional areas for this design are presented in Table 2. Additionally, Figure 9 shows the reference topology obtained by Shakya et al. [33], Wu and Tseng [44] for a 12-node problem. The bone remodeling algorithm was run for this reference topology, and the results (Reference Topology column) given in Table 2 were generated. The results obtained with the bone remodeling algorithm presented in this study yield a structure that is only slightly lighter than the literature results. Although the problems in Shakya et al. [33], Wu and Tseng [44] address displacement constraints (allowable displacement value ±50.8 mm), it is understood that these constraints are not active.

Table 2 shares the design of the truss structure obtained at the end of Phase II of this study in the last column, whose topology is presented in Figure 8. Although the topology obtained in this study differs from the “reference topology”, both designs have the same weight. Additionally, in both designs, the vertical displacements at the three loaded nodes are −30.4809, −36.5771, and −30.4809 mm, respectively, moving from left to right. This indicates that the two designs with different topologies have the same rigidity. The total deformation energy for both designs is U=4338.73 J. Although one topology is statically determinate and the other is statically indeterminate, it is interesting and a unique finding that they have the same weight and rigidity. In this study, both solutions obtained with the bone remodeling algorithm adapted for truss structures (the last two columns in Table 2) are within the stress limits for all bars except bars 20 and 21. This finding demonstrates that the bone remodeling algorithm used in this study can successfully produce results. Bars 20 and 21, which have zero stress and cross-sectional areas equal to the lower limit, are included in the system because they are necessary for kinematic stability. Ultimately, the 12-node truss problem analyzed here serves as a representative model for modular roof trusses or bridge girders commonly found in industrial steel structures, highlighting the practical significance of these optimal findings.

### 3.2. 247-Node Fully-Connected GS

**Figure 10:**
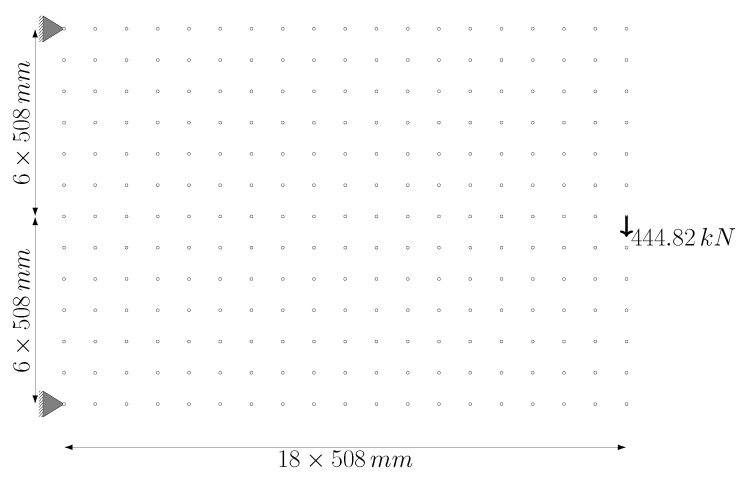
247-node ground structure nodes, supports and loading conditions (where the vertical arrow represents the applied point load).

In the second example problem, the system consists of 247 nodes arranged at equal intervals of 18 in the horizontal direction and 12 in the vertical direction. The intervals in both directions are 508 mm. The ground structure obtained by connecting each node point with bars which are m=30,381 bars, in total. The degree of freedom of the structure is p=490, and the degree of indeterminacy is r=29,891. The material elastic modulus E=68.95 MPa, unit volume weight, ρ=27.14 kN/m^3^, and stress lower and upper limit values are used as $\sigma^{l}=-34.48$ MPa and $\sigma^{u}=34.48$ MPa, respectively. The cross-sectional areas having lower limit value $A^{l}=58.06$ mm^2^ and the upper limit value $A^{u}=22,580.6$ mm^2^. The support nodes, loading condition, and dimensions are given in Figure 10.

**Figure 11:**
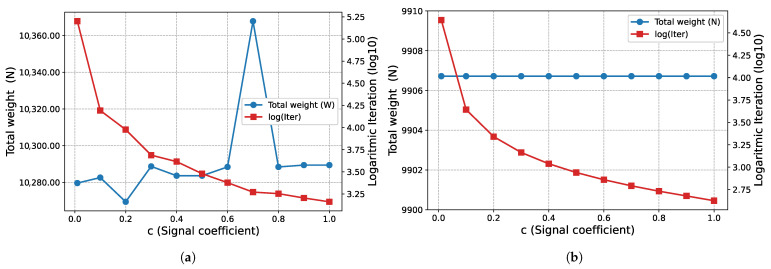
Influence of parameter c on the optimal weights and the required number of iterations in the 247-node GS: (**a**) Phase I; (**b**) Phase II.

The proposed optimization algorithm is executed over a wide range of signal coefficients to evaluate its performance and stability on this large-scale ground structure. This parametric study aimed to assess the effect of signal coefficients on the final topological results across both phases. The inverse relationship between the signal coefficient *c* and the convergence rate, as illustrated in Figure 11, is similar in the second example problem. While, the total weight (*W*) values obtained at the end of Phase I (Figure 11a) fluctuate within a narrow range, they remain constant at the end of Phase II (Figure 11b) for all examined *c* values. Regarding the Phase I results, the average weight value obtained for all analyzed *c* values is 10,291.90 N and the standard deviation is 25.86 N. The minimum and maximum weight values recorded are 10,269.4 N and 10,367.8 N, respectively.

**Figure 12:**
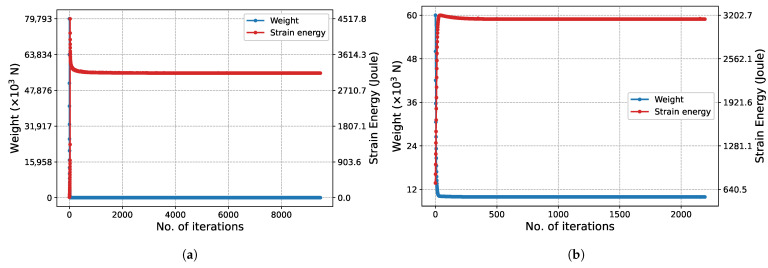
Convergence history of structural weight and strain energy for the 247-node GS: (**a**) Phase I; (**b**) Phase II.

The convergence characteristic of the algorithm is also observed in Figure 12 for the 247-node GS problem, which has a much larger design space. Although the convergence process naturally requires more iterations due to the enormous increase in the number of design variables, the characteristic form of the strain energy, with a transition peak followed by asymptotic convergence to equilibrium, is preserved.

**Figure 13:**
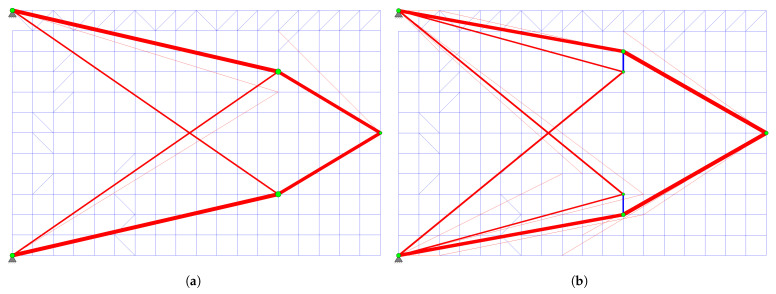
Phase I results for the 247-node GS, grouped by the common ground structures established for Phase II (blue: primary members; red: redundant members): (**a**) specific topology obtained for c=0.70; (**b**) representative topology for ten *c* values (excluding c=0.70).

In this example problem, although the obtained structure weights differ for each distinct value of the signal coefficient *c* (Figure 11), the resulting representative topologies form two groups. The design resulting at the end of Phase I for the signal coefficient c=0.7 is shown in Figure 13a. The representative design for other *c* values is presented in Figure 13b.

**Figure 14:**
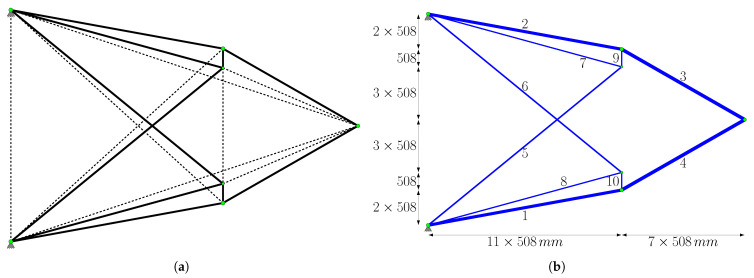
Reconstructed ground structure and the resulting optimum topology for Phase II: (**a**) Generated ground structure for Phase II (shown in black); (**b**) Final topology for the 247-node GS after Phase II optimization (blue: primary members).

The representative topology obtained at c=0.2 (Figure 13b) is selected to initiate Phase II, as it yielded a more efficient structural weight (10,269.40 N) compared to the result for c=0.7 (10,367.8 N). Based on the filtering process described in Section 2.3, the ground structure shown in Figure 14a is established. The reconstructed ground structure consists of 18 bars in total. Among these, 10 are “primary candidate members” (solid lines) identified at the conclusion of Phase I, while the other 8 elements (dashed lines) have been strategically included to ensure both kinematic stability and to recover elements that may have been accidentally excluded during filtering, thereby ensuring that the necessary bars are present in the system for the final optimal solution.

When the optimal design of this reconstructed ground structure is sought using the bone remodeling algorithm, the final topology presented in Figure 14b is achieved. The corresponding cross-sectional areas and the final structural weight of 9906.72 N are detailed in Table 3. It is observed that the bars identified as “primary candidate members” constitute all the members in this optimal topology.

Although the optimal structural weight reported by Shakya et al. [33] is 9786.11 N with a different configuration, the results of this study show a minimal difference of only 1.2%, validating the effectiveness of the proposed method. Shakya et al. [33] reported that the displacement constraint ($\delta_{a}=\pm 15.24$ mm) is not active for their final topology. Although the current study yields a different structural layout than the reference, the results agree on the inactivity of the displacement limit. The recorded maximum vertical displacement of −14.1416 mm at the loaded node remains well within the allowable range.

**Figure 15:**
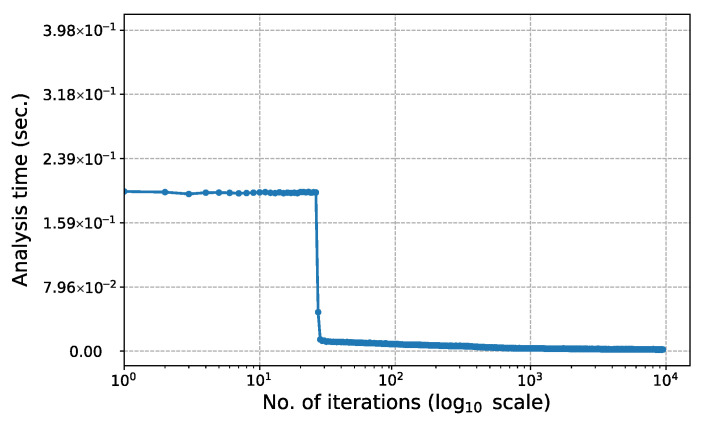
Evolution of analysis time relative to the number of iterations for the 247-node ground structure (Phase I).

Figure 15 shows the change in computation time for each iteration of the 247-node ground structure in Phase I (c=0.2). To more clearly highlight the performance characteristics in the initial phase of the analysis, the horizontal axis is plotted on a $\log_{10}$ scale. As seen in the graph, the time spent on each structure analysis remains constant until approximately the 25th to 30th iteration, after which a sharp decrease in analysis time is observed. This sudden change occurs because the bone remodeling algorithm simultaneously removes a large number of elements from the structure. The technical reasons behind this decrease in time and its relationship with the change in the matrix structure are discussed in detail in the Discussion section.

### 3.3. 2D Michel Truss

To determine the degree to which the developed method approximates theoretical solutions, a design problem with an analytical counterpart is examined. The numerical model is prepared using the optimal volume criteria defined by Michell, and is detailed below.

The design area of the problem in question has dimensions of 20×250 mm (5000 mm) in the *x*-direction and 8×250 mm (2000 mm) in the *y*-direction. As boundary conditions, the structure is fixed at the start and end points (0 mm and 5000 mm) at the bottom edge level (y=0). The external loading is applied as a single force at the midpoint of the bottom edge (2500 mm). In a similar study in the literature Lai et al. [45], a system consisting of 3116 bars is constructed using 189 nodes and third-level connections. In contrast, the present study adopted a fully-connected ground structure approach to represent the design space more comprehensively; accordingly, a total of 17,766 bars are included in the analysis, considering all possible interactions between nodes.

**Figure 16:**
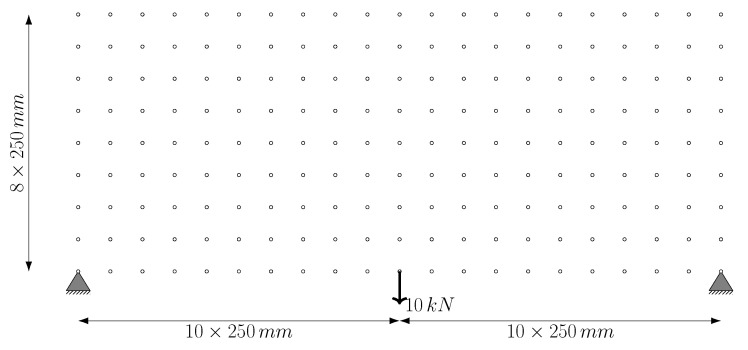
21 × 9 Michel ground structure nodes, supports and loading conditions (where the vertical arrow represents the applied point load).

Figure 16 shows the node locations, supports, and loading conditions. The analytical solution for the trusses is given by Michell [46] as follows:

(33) $$V_{analitacal}=P\left(\frac{L_{x}}{2}\right)\left(\frac{1}{2}+\frac{\pi }{4}\right)\left[-\frac{1}{\sigma^{l}}+\frac{1}{\sigma^{u}}\right]$$

Here, $L_{x}$ and $L_{y}$ denote the dimensions of the design area along the *x* and *y* directions, respectively.

In the analyses performed, the material’s elastic modulus, $E=2.06\times 10^{5}$ MPa, and stress limits of ±215 MPa are used. Lai et al. [45] gives the lower and upper limit values of the cross-sectional area as $A^{l}=10^{-6}$ mm^2^ and $A^{u}=10^{6}$ mm^2^, respectively. However, in this study, the lower limit value of the cross-sectional area is used as $A^{l}=0.1$ mm^2^ to facilitate the removal of redundants.

**Figure 17:**
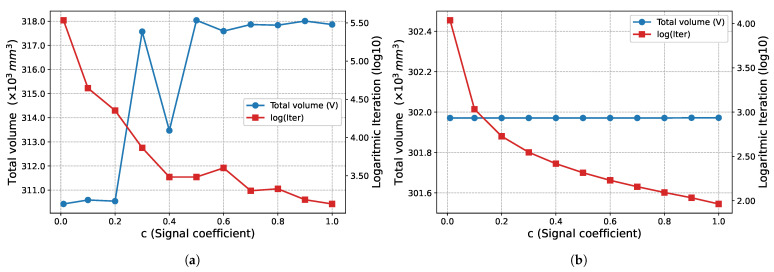
Influence of parameter c on the optimal weights and the required number of iterations in the 2D Michell truss: (**a**) Phase I; (**b**) Phase II.

The proposed optimization algorithm is executed with various signal coefficients to evaluate its consistency for this specific problem. In this parametric study the impact of the signal coefficient on convergence behavior across both phases is analyzed. The effect of the signal coefficient *c* on the number of iterations, as illustrated in Figure 17, is similar in this example problem as in other example problems. While the total volume (*V*) values obtained at the end of Phase I (Figure 17a) are clustered around the average value of 315,435.55 mm^3^ and the standard deviation of 3412.52 mm^3^, they remain constant at 301,971.0 mm^3^ at the end of Phase II (Figure 17b) for all examined *c* values. Regarding Phase I, the lowest volume value recorded in the examined parameter range is measured as 310,427 mm^3^, while the highest value is 318,037 mm^3^.

**Figure 18:**
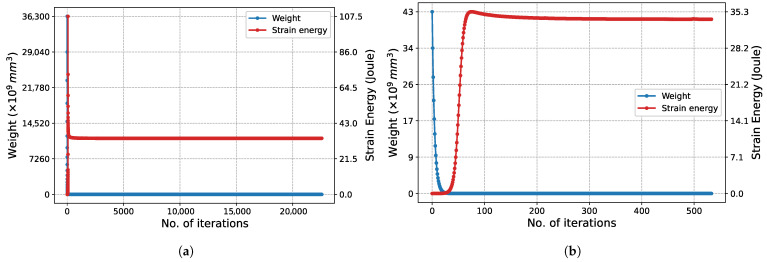
Convergence history of structural volume and strain energy for the 2D Michell truss: (**a**) Phase I; (**b**) Phase II.

The characteristic convergence behavior of the method is in perfect agreement with the results for the 2D Michell truss structure presented in Figure 18. In this problem, particularly in Phase I (Figure 18a), due to the high total number of iterations, the peak in the strain energy is observed as an instantaneous jump. In contrast, in Phase II (Figure 18b), this transition is observed to occur with a more gradual and smooth trend. This difference is based on the significant narrowing of the design space in Phase II and the continuation of optimization only through the limited number of critical bar elements carrying the load.

**Figure 19:**
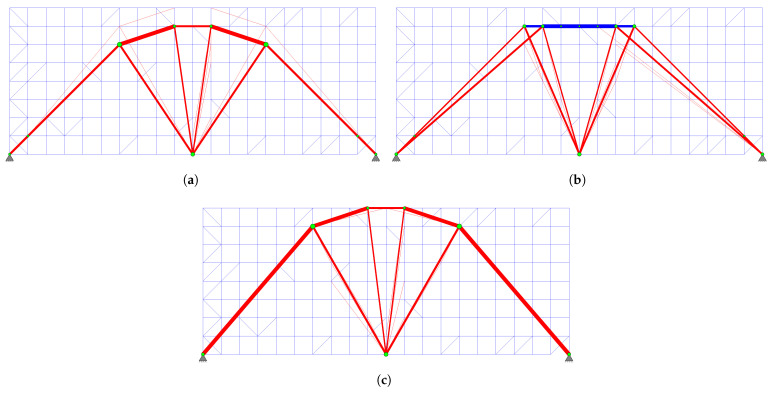
Phase I results for the 2D Michell truss (blue: primary members; red: redundant members), categorized by the common ground structures established for Phase II: (**a**) representative results for c∈{0.01,0.1,0.2}; (**b**) representative results for c∈{0.3,0.5,0.6,0.7,0.8,0.9,1.0}; (**c**) result for c=0.4.

**Figure 20:**
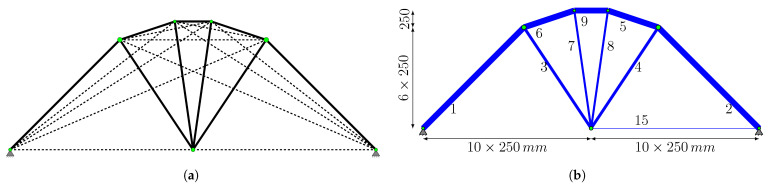
Reconstructed ground structure and the resulting optimum topology for Phase II: (**a**) generated ground structure for Phase II (shown in black); (**b**) final topology for the 2D Michell truss after Phase II optimization (blue: primary members). Member indices follow the generated ground structure numbering, with gaps representing members removed during optimization.

When the topological elimination steps detailed in Section 2.3 are applied, the structures obtained at the end of Phase I for various signal coefficients *c* are grouped into three main categories (Figure 19). The analysis revealed that the results for c∈{0.01,0.1,0.2} offer the lowest structural weights; consequently, the representative topology from this group (Figure 19a) is selected as the basis for Phase II. Accordingly, the ground structure shown in Figure 20a is reconstructed, comprising 20 bars in total. Among these, 9 of them are “primary candidate members” (solid lines), while the other 11 members (dashed lines) are strategically included not only to ensure kinematic stability but also to recover any members that might have been inadvertently excluded during filtering and to provide all necessary bars required for the final optimal solution.

Phase II is completed by running the bone remodeling algorithm with the parameter c=0.2 on this reconstructed ground structure. The optimization results obtained are presented in Table 4. The final topology consists of 10 bars, and it is determined that the remaining 9 bars, except member 15, reached their stress limits. These 9 bars are included in the “primary candidate members” list. Although not included in the list, it is observed that member 15, which is included in the system using the Gauss–Jordan operation, is a ‘zero member’ that provides kinematic stability and that its cross-sectional area remains at the defined lower limit value. The maximum vertical displacement, reflecting the structure’s stiffness performance, is recorded as −6.7704 mm at the loading point.

The analytical solution provided by Michell [46] gives the structure volume as V=298,930 mm^3^. At the end of Phase II, the solution obtained using the bone remodeling algorithm is 301,971 mm^3^. There is a difference of %1.02 between the solution obtained in this study and the analytical solution. The result reported by Lai et al. [45] in the current literature has a higher topological complexity than that obtained in this study, but remains close to the analytical solution at 1.19% ($3.024\times 10^{5}$ mm^3^). In this study, the fact that the proposed method can produce a solution that is both close to the analytical solution and simpler than those in the recent literature demonstrates its numerical accuracy and potential to yield optimised forms advantageous for manufacturing.

**Figure 21:**
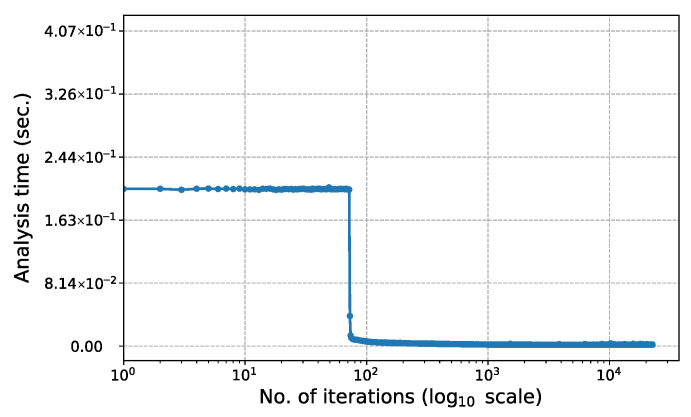
Evolution of analysis time relative to the number of iterations for the 2D Michell truss (Phase I).

Figure 21 shows the change in computation time for each iteration of the 2D Michell truss structure in Phase I (c=0.2). The horizontal axis is plotted on a $\log_{10}$ scale to more clearly highlight the performance characteristics at the Phase I. It can be observed that the structure analysis times remain nearly constant up to approximately the 75th iteration, but then decrease sharply from this point onwards. This situation arises because the bone remodeling algorithm removes a large number of elements from the system. The technical reasons behind this decrease in time and its relationship with the change in the matrix structure are discussed in the Discussion section.

### 3.4. 3D Fully Connected GS

**Figure 22:**
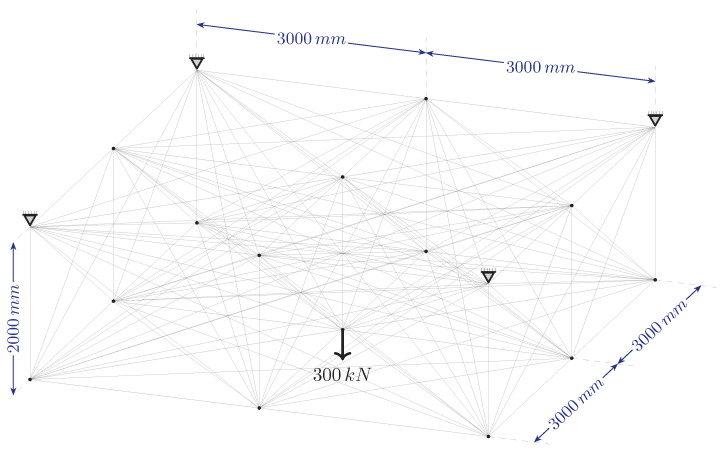
The 3D fully-connected ground structure nodes, supports and loading conditions.

The final problem examined is a three-dimensional ground structure comprising 18 nodes [33]. Figure 22 shows the geometric dimensions, supported nodes and load conditions of the structure. This fully connected structure has 153 elements. In numerical analyses, the elastic modulus of the material is taken as 200 GPa and its unit volume weight as 77.0 kN/m^3^. The allowable lower and upper stress limits for all members are set at ±150 MPa. The lower limit of the cross-sectional areas is given as 0 by Shakya et al. [33]. However, since the primary forces cannot be eliminated in this study, the lower limit value of the cross-sectional area for the primary forces is taken as $A^{l}=0.1$ mm^2^. The lower limit values for redundants are 0, and the upper limit value for all bars is $A^{u}=2000$ mm^2^.

**Figure 23:**
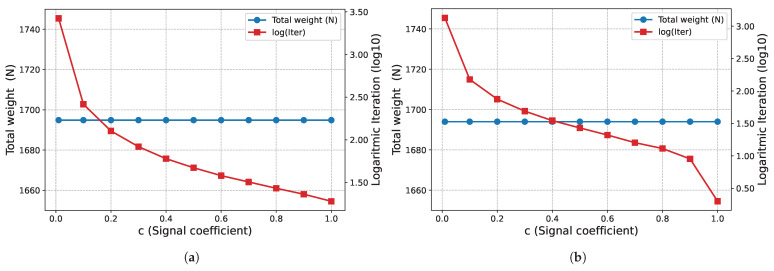
Influence of parameter c on the optimal weights and the required number of iterations in the 3D truss: (**a**) Phase I; (**b**) Phase II.

The 3D truss structure results presented in Figure 23 show that the algorithm continues its characteristic behavior observed in previous examples in this case as well. The total structure weight remains constant for all *c* values examined, with values of W=1694.87 N at the end of Phase I (Figure 23a) and W=1694 N at the end of Phase II (Figure 23b). As observed in other problems and as expected, the number of iterations required for convergence increases logarithmically as the signal coefficient decreases.

**Figure 24:**
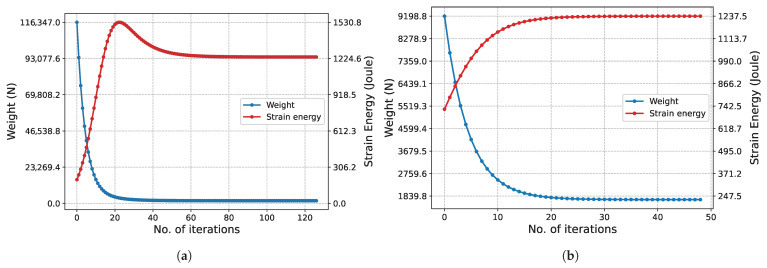
Convergence history of structural weight and strain energy for the 3D truss: (**a**) Phase I; (**b**) Phase II.

The convergence history of the 3D truss problem considered as the final case is presented in Figure 24. In the optimization process initiated with 153 bars in Phase I, it is observed that the characteristic ‘peak and equilibrium’ form of the strain energy is maintained in the 3rd dimension as well. Unique to this problem, the optimal topology has already been achieved at the start of Phase II. Therefore, the algorithm attempts to determine the appropriate cross-sectional areas within a given topology. Since all bars have the maximum cross-sectional area ($A^{u}$) at the start of the algorithm, the structure is at its most rigid state, and the strain energy is at its lowest level. As the cross-sectional areas decrease, the structure weight decreases, while the strain energy asymptotically increases to reach the equilibrium value, exhibiting a very smooth transition.

**Figure 25:**
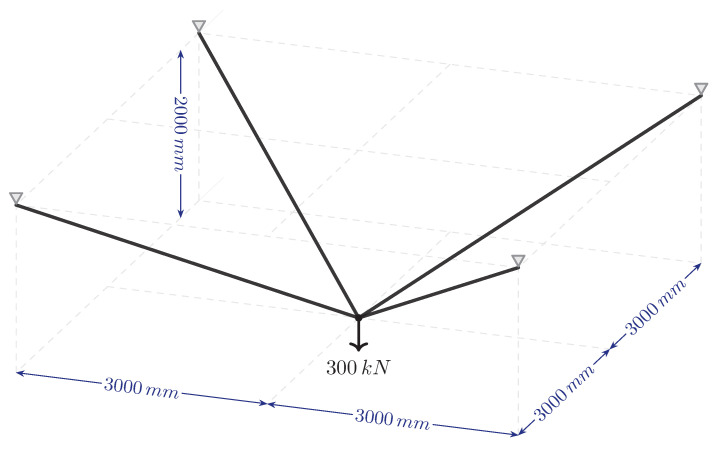
Final topology for the 3D fully-connected ground structure after Phase II. optimization.

In the final topological structure that provides the connection between the load and support points, the cross-sectional area of the four bars is equal and determined to be 1172.6 mm^2^ (Figure 25). Although the obtained topology is statically indeterminate, all bar stresses are at their limit values. The total weight of the structure is 1694 N. A vertical displacement of −8.2500 mm is observed at the point of load application. The results obtained from this study are in complete agreement with the design reported by Shakya et al. [33].

## 4. Discussion

In structural topology optimization, the selection of the analysis method is critical for both computational efficiency and the stability of the results. While the displacement method is the standard analysis tool, the fundamental objective in topology optimization problems is to remove unnecessary elements. Determining which elements are necessary cannot be easily established solely by examining whether they carry force or not. As shown in the examples considered in this study (Pr. 1 and Pr. 3), there are bars carrying no forces but they are necessary in the system for kinematic stability. When the displacement method is used, instead of completely removing the cross-sectional areas to prevent stability problems, it is allowed to have very small cross-sectional areas. However, in this case, the system may deviate from the true optimum value due to the bars that appear to exist in the system [32]. Apart from the displacement method, the other two accepted analysis methods are the force method and the integrated force method (IFM). The literature contains studies using genetic algorithms and the force method for the topology optimization of truss structures [47,48,49,50,51]. However, in all of these studies, the degrees of indeterminacy of the examined examples are smaller than the degrees of freedom (p>r). In this case, the force method is advantageous for analysis time because it yields a smaller set of equations. On the other hand, since fully connected ground structures initially contain $\frac{n\times (n-1)}{2}$ bars, the number of redundant forces (*r*) reaches huge values depending on the number of nodes. This situation creates a fundamental limitation by increasing the computational cost of the force method and IFM, which has even more unknowns [52,53,54]. The PFB approach stands out at this point for its structure, which makes the equation size independent of the degree of indeterminacy.

The proposed PFB approach is designed to overcome these limitations and to ensure kinematic stability in large-scale systems. Performing structural analysis using the PFB approach is a proposed solution to the challenge of guaranteeing kinematic stability, one of the most fundamental problems in the topology optimization of truss structures. Thanks to this approach, truss structure members can be divided into two groups: those that can be “safely removed” from the system and those that “cannot be removed” (primary structure). Although this advantage is also present in the force method, the rapid increase in the degree of indeterminacy in fully connected ground structures makes it a disadvantage. On the other hand, the inability to remove the primary structure bars can be an obstacle to fully reaching the final topology. To overcome this problem, a two-phase solution strategy is developed in the study. Critical information about the final topology is obtained by determining the required nodes and bars through the filtering operation between the two phases. In Phase II, a more refined ground structure, a subset of the initial structure that represents the load paths, is established. At this phase, the bars that should be present in the final topology are labeled as “primary candidate members” and included in the primary structure. Thus, by working with a much smaller subset, the ground structure’s topology can be obtained in a very short time.

The strategic advantages offered by this method are directly supported by reduced processing costs. As a characteristic of the “primary force-based” analysis method described in Section 2.1, removing bars from the system does not change the size of the equation set (degrees of freedom); however, the time required to set up and solve the system matrix is reduced. This situation is directly related to the performance of the Cholesky decomposition used in solving the system of equations. The bars removed from the system as a result of the iterative elimination process cause the skyline profile of the system matrix to decrease. As the profile height decreases, the computational cost of the decomposition process decreases, allowing the structural analysis to be completed much more quickly. This situation is directly related to both the ability to completely remove redundant forces from the system without worrying about kinematic stability issues in the proposed analysis method and the performance of the bone remodeling algorithm.

The practical results of these mathematical improvements in the calculation process are presented in Table 5 for different problem types and signal coefficients. Phase I durations are documented for all eleven *c* values. In contrast, the Phase II results and the final total analysis times are reported for c=0.20 as a representative case. While all *c* values were tested to ensure consistency, Phase II durations remained negligible across the entire range, consistently falling below 0.2 s. At this millisecond scale, minor fluctuations are primarily due to transient system-level background processes rather than algorithmic complexity. This efficiency proves that the refined ground structure from the end of Phase I successfully streamlines the search space for the final optimization.

The computational speed of the proposed method demonstrates a significant advantage over similar studies in the literature. Examining the total times obtained for the parameter c=0.2 in the proposed strategy reveals the following values: 0.0050 s for Problem 1, 35.92 s for Problem 2, 60.50 s for Problem 3 and 0.0073 s for Problem 4. The times reported in the literature for these problems are as follows: (Problem 1: 3.5 s; Problem 2: 399.1 s; Problem 4: 7.1 s) [33], and (Problem 3: 270 min) [45]. Notably, Problem 3 is solved in approximately 60 s in this study. In contrast, the result obtained by Lai et al. [45] in 270 min (16,200 s) demonstrates the efficiency of combining the bone remodeling algorithm with the PFB approach in complex, large-scale systems. Even accounting for hardware differences, this order-of-magnitude difference in time confirms the algorithmic superiority of the methodology.

The slight 1.02% difference between the numerical results obtained in Problem 3 and Michell’s analytical solution (298,930 mm^3^) is attributed to the discretization of the ground structure’s geometry. Michell’s theory assumes an infinite network of bars perfectly aligned with the principal stress trajectories. In contrast, the current numerical model is naturally constrained by predefined node coordinates and bar connections. If the node arrangement had been specifically adapted to the geometry of the Michell structure, the numerical result would be expected to converge even closer to the theoretical minimum. Nevertheless, achieving such a close result within a finite ground structure confirms the high accuracy and efficiency of the proposed bone remodeling algorithm.

The results obtained for Problem 1 (12-node truss) are noteworthy for demonstrating the optimization algorithm’s flexibility in the design space. Compared with the reference study in the literature, although the proposed algorithm produces a visually different topology, it achieves exactly the same values for total weight and system stiffness as the reference design.

This high degree of accuracy in numerical models is also closely related to the parametric sensitivity of the underlying biological adaptation mechanism. When examining the effects of the signal coefficient *c*, a clear inverse correlation between calculation speed and solution quality is observed. This situation directly corresponds to the principles of mass change in the bone matrix, which result from chemical reactions that are pretty slow and depend on stress. Figure 5, Figure 11, Figure 17 and Figure 23, a decrease in the *c* coefficient increases the total number of iterations required for convergence but enables the algorithm to reach lower-weighted results, especially for complex problems with a large search space. This situation can be interpreted as low signal coefficients increasing the algorithm’s “search” capability in the design space, thereby reducing the risk of getting stuck in local minima that high speed could cause.

This algorithm’s research capability necessitates determining the most suitable operating parameters for different problem types. The fluctuations in the data in Figure 11 (247-node GS) and Figure 17 (2D Michell truss) confirm that the algorithm converges early at high *c* values, resulting in suboptimal topologies. In contrast, at low *c* values, the algorithm can obtain lighter configurations by more accurately simulating the speed at which the signal level adapts to the structure. This sensitivity in Phase I arises because large-scale, fully connected ground structures encompass a vast design space with numerous topological possibilities. The results for small-scale problems (12-node truss and 3D GS) show that Phase I is significantly less sensitive to the *c* value, exhibiting a stability similar to Phase II (Figure 5a and Figure 23a). However, as the process enters Phase II, the algorithm operates on a significantly refined structure. This streamlined search space leads to the consistency observed across all *c* values. Based on the analysis, setting *c* to 0.1 or 0.2 is recommended to balance solution quality and iteration count. While high *c* values, such as 1.0, produce fast results, these should be avoided as they may affect the efficiency of the final design. The suggested *c* coefficient values presented here are based on the results derived from the problems examined. Therefore, it would be inappropriate to draw a universal inference. It should be remembered that these values may vary depending on the specific requirements of the problem.

## 5. Conclusions

This study successfully created a computationally efficient optimization framework for large-scale trusses by integrating a primary force-based analysis approach with biomimetic bone remodeling principles. Addressing the research objectives outlined in the introduction, the following results were obtained:

- The developed PFB analysis approach effectively eliminated the numerical instabilities typically encountered in the displacement method. This approach combines the kinematic stability guarantee of the force method with the reduced system equation size advantage of the displacement method. Kinematic stability was guaranteed by preserving the initial primary structure throughout the process. This approach was made possible by removing only the redundant members.
- The application of an optimization rule based on Wolff’s Law has demonstrated that biological adaptation principles can be successfully used as an effective engineering approach for truss structures. Element cross-sections ($A_{j}$) were updated using stress-based stimulators equivalent to SED in truss systems. This approach provided a reliable mechanism for mimicking the natural efficiency of bone remodeling in discrete systems.
- The proposed two-phase strategy has played a fundamental role in overcoming the topological constraints imposed by the non-removable elements in the primary structure of the PFB method. The transition between Phase I and Phase II was performed using energy-dense node and bar filtering. These processes successfully refined the design space, enabling the attainment of the final optimal topology. Consequently, the entire optimization was completed without compromising the system’s structural integrity.
- Numerical comparisons with existing literature validated the precision of the proposed method. The algorithm demonstrated high convergence performance, achieving results within 1.02% of analytical Michell solutions while providing a 10 to 250-fold speedup compared to previous studies.

Truss structures serve as fundamental modeling elements across diverse fields, ranging from industrial facilities and aerospace vehicles to micro-scale structures. Although the PFB approach was developed specifically for truss elements in this study, it can be extended to frames or other element types, indicating its potential for broader structural analysis applications. Consequently, the proposed methodology is also highly applicable to the design and optimization of various truss-like structures. Practical applications include the development of lightweight sandwich panels for aerospace components and lattice scaffolds for biomedical bone implants.

## Figures and Tables

**Table 1:** Control parameters used in the optimization process.

Parameter	Symbol	Value
Maximum number of iterations	$t_{max}$	500,000
Weight convergence tolerance	$\varepsilon_{w}$	$10^{-5}$
Limit for normalized constraint violations	$\varepsilon_{v}$	$10^{-5}$
Cumulative energy contribution ratio	$\gamma_{cum}$	0.97
Local energy averages ratio	$\eta_{ratio}$	0.03

**Table 2:** Comparison of optimized cross-sectional areas (mm^2^) and total weights with literature results.

Member	Shakya et al. [33]	Deb and Gulati [43]	Wu and Tseng [44]	Reference Topology (Figure 9)	This Study
$A_{1}$, $A_{2}$	967.740	969.030	967.740	967.701	967.701
$A_{3}$, $A_{4}$	–	32.903	–	–	–
$A_{5}$, $A_{6}$	684.515	685.805	683.870	684.268	436.876
$A_{7}$, $A_{8}$	–	32.903	–	–	–
$A_{9}$, $A_{10}$	684.515	684.515	683.870	684.268	766.732
$A_{11}$, $A_{12}$	483.870	484.515	483.870	483.851	542.161
$A_{13}$, $A_{14}$	161.290	161.935	161.290	161.284	102.973
$A_{15}$, $A_{16}$	360.644	360.644	360.644	360.641	230.254
$A_{17}$, $A_{18}$	–	33.548	–	–	–
$A_{19}$	645.160	648.386	645.160	645.134	–
$A_{20}$, $A_{21}$	32.258	–	32.258	32.258	32.258
$A_{22}$, $A_{23}$	–	–	–	–	411.891
$A_{24}$, $A_{25}$	–	–	–	–	260.773
$A_{30}$	–	–	–	–	233.243
Weight (N)	859.396	874.280	859.392	859.213	859.213

**Table 3:** Optimal cross-sectional areas and total weight for the 247-node GS (areas in mm^2^).

$A_{1}$, $A_{2}$	$A_{3}$, $A_{4}$	$A_{5}$, $A_{6}$	$A_{7}$, $A_{8}$	$A_{9}$, $A_{10}$	Weight (N)
11,476.60	13,005.00	5210.45	4179.95	4399.28	9906.72

**Table 4:** Optimal cross-sectional areas and total weight for the 2D Michell truss (areas in mm^2^).

$A_{1}$, $A_{2}$	$A_{3}$, $A_{4}$	$A_{5}$, $A_{6}$	$A_{7}$, $A_{8}$	$A_{9}$	$A_{15}$	Volume (mm^3^)
32.889	15.254	33.429	10.678	33.223	0.100	301,971.0

**Table 5:** Problem analysis times.

			Time (s)			
		*c*	12-Node Truss	247-Node GS	2D Michell Truss	3D GS
Phase I	DPS ^1^	-	0.0001	4.4936	1.5398	0.0005
		0.01	0.0481	570.1840	1205.7800	0.0984
		0.10	0.0030	53.6888	120.5500	0.0096
		0.20	0.0015	31.4155	58.9533	0.0049
		0.30	0.0010	17.2009	26.2603	0.0030
		0.40	0.0008	13.8836	14.4841	0.0023
		0.50	0.0007	10.2620	11.6717	0.0023
		0.60	0.0006	8.4711	12.9893	0.0015
		0.70	0.0005	5.5397	7.7014	0.0015
		0.80	0.0004	6.1476	7.1142	0.0012
		0.90	0.0004	4.8630	5.4206	0.0010
		1.00	0.0003	4.0163	3.6266	0.0011
Phase II	DPS ^1^	-	$2.60\times 10^{-5}$	$1.10\times 10^{-5}$	$1.10\times 10^{-5}$	$0.50\times 10^{-5}$
		0.20	0.0034	0.0115	0.0029	0.0019
Total time for c=0.20			0.0050	35.9206	60.4960	0.0073

^1^ DPS: Time Elapsed to Determine the Primary Structure.

## Data Availability

The original contributions presented in this study are included in the article. Specifically, the algorithmic logic is detailed via pseudo-codes within the methodology section. The full source code is not publicly available due to its integration into a broader proprietary software ecosystem but may be made available from the corresponding author upon reasonable request.
